# Evidence of the circulation of avian metapneumovirus in domestic backyard chickens in Eastern Saudi Arabia in 2019

**DOI:** 10.14202/vetworld.2023.1246-1251

**Published:** 2023-06-08

**Authors:** Abdullah I. A. Al-Mubarak, Jamal Hussen, Mahmoud Kandeel, Anwar A. G. Al-Kubati, Baraa Falemban, Maged Gomaa Hemida

**Affiliations:** 1 Department of Microbiology, College of Veterinary Medicine, King Faisal University, Saudi Arabia; 2 Department of Biomedical Sciences, College of Veterinary Medicine, King Faisal University, Al-Hofuf, Saudi Arabia; 3 Department of Pharmacology, Faculty of Veterinary Medicine, Kafrelsheikh University, Kafrelsheikh, Egypt; 4 Department of Veterinary Medicine, Faculty of Agriculture and Veterinary Medicine, Thamar University, Thamar, Yemen; 5 Department of Veterinary Biomedical Sciences, College of Veterinary Medicine, Long Island University, USA

**Keywords:** antibodies, chickens, pigeons, Saudi Arabia

## Abstract

**Background and Aim:**

Avian metapneumovirus (aMPV) is a recently discovered respiratory virus in chickens. Avian metapneumovirus has been linked to respiratory syndromes, reproductive failure in affected chickens and turkeys, swollen head syndrome in chickens, and rhinotracheitis in turkeys. Wild birds are considered potential reservoirs of aMPV, particularly aMPV-C. However, little is known about the prevalence of aMPV in Saudi Arabia. Considering the relevance of backyard chickens in the transmission and sustainability of certain avian viral diseases, this study aimed to assess aMPV exposure in backyard chickens and wild birds circulating near selected locations.

**Materials and Methods:**

We collected 368 serum samples from unvaccinated backyard chickens in ten locations in Eastern Saudi Arabia. Furthermore, we collected 78 serum samples from species of free-ranging birds belonging to the Columbidae family, such as pigeons and doves, captured from the same areas. Using commercial enzyme-linked immunosorbent assay kits, we tested the sera of domestic backyard chickens and wild birds for antibodies against aMPV.

**Results:**

Our results showed that 74/368 birds were positive for aMPV-related antibodies. Conversely, none of the tested wild birds seroconverted to aMPV.

**Conclusion:**

The antibody titers detected in the backyard chickens suggested recent exposure to aMPV. Considering these results, further large-scale serological and molecular studies are needed to evaluate the prevalence of aMPV in these birds and characterize the circulating strains of aMPV in this region.

## Introduction

Respiratory avian viral diseases cause extensive economic losses [1]. This might be attributable to high morbidity and mortality and poor growth rates according to the clinical signs associated with infection. Backyard chickens might play a key role in the local circulation of diseases, particularly viral diseases [2], due to insufficient biosecurity measures and potential close contact with wild birds [3–5]. Furthermore, the lack of specific vaccination programs represents another burden of backyard chicken farming [5]. Although there are sanitary risks associated with this system, raising birds in backyards is a common practice in many countries, and it is usually characterized by the association of different species of birds of various ages, such as chickens, geese, quails, and turkeys, kept together in a condensed space, in which they share habitats, food, and water sources.

**Figure-1:**
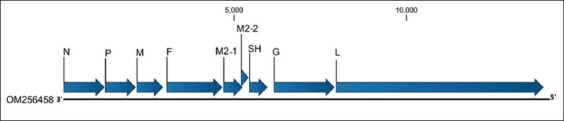
The genome structure and organization of avian metapneumovirus. The genome consists of a single linear of negative-sense RNA molecule. The genome encodes eight genes which produce nine proteins (nucleocapsid (N), phosphoprotein (P), matrix protein (M), fusion protein (F), two overlapped accessory matrix proteins ((M1 and M2), small hydrophobic (SH), the attachment protein (G), and the RNA-dependant RNA polymerase (L)). The genome is flanked with a 3’ leader and a 5’ trailer.

Wild birds can also approach these backyards for food and water; therefore, this environment favors the emergence and transmission of viral, bacterial, or parasitic diseases between domestic and wild birds [6]. As an example, outbreaks of several viruses, such as highly pathogenic avian influenza virus, Newcastle diseases virus, infectious bursal diseases virus, avian encephalomyelitis virus, Marek’s disease virus, and poxvirus, have been reported in backyards in Egypt, Turkey, England, Italy, and Greece [7–11]. Among viral diseases, avian metapneumovirus (aMPV) contributes to high economic losses in the poultry industry. The aMPV genome is 13 kb in size and encodes eight proteins (Figure-1): nucleoprotein, phosphoprotein, matrix protein, fusion protein, SH protein, glycoprotein, and polymerase. From an epidemiological perspective, aMPV has been detected in various species of wild and domestic birds, particularly broiler chickens and turkeys [12]. Infection is usually associated with respiratory signs in birds according to the species’ susceptibilities. In particular, aMPV is the causative agent of turkey rhinotracheitis and is associated with swollen head syndrome (SHS) in chickens [13]. The virus can cause marked decreases in egg production associated with *Escherichia coli* infection in layer chickens. Both pathogens are responsible for egg malformation. In some cases, chickens may be infected and seroconverted to aMPV without showing any obvious clinical signs [14]. Based on the most recent classification, the International Committee of Taxonomy and Nomenclature of Viruses recently classified aMPV and human metapneumovirus under the genus *Metapneumovirus* in the family Pneumoviridae. aMPV has been further classified into four subgroups (A–D) [15] based on antigenic and genetic differences, and two divergent strains were recently found in domestic gulls and monk parakeets (*Myiopsitta monachus*) [16, 17]. Since its emergence in South Africa, aMPV has continued to circulate in domestic birds in many countries globally [12, 18–20]. Unfortunately, there is no current report on the prevalence of aMPV in chickens in Saudi Arabia or the Gulf region. The presence of some wild or migratory species in backyards could be responsible for the emergence and transmission of some pathogens [6]. This transmission can occur bidirectionally from wild birds to domestic birds or vice versa [6]. The backyard business is growing in many countries, and it is considered a significant risk factor for the emergence of some pathogens due to non-existent or insufficient biosecurity standards [6].

Thus, active surveillance for common pathogens, particularly viral diseases, among backyard birds is essential for monitoring the emergence of new pathogens or outbreaks of known pathogens. Given this background, the main goal of the present study was to conduct a serological survey of aMPV in unvaccinated backyard chickens and wild birds in close contact with those chickens to increase knowledge about their exposure to aMPV in Saudi Arabia.

This study examined the history of exposure of backyard chickens and migratory birds to aMPV. The findings of this study will encourage additional surveillance studies to clarify the exposure/immune status of backyard chickens and other wild birds to viruses, particularly aMPV.

## Materials and Methods

### Ethical approval

The chicken and wild birds sampling activities carried out in this study were conducted as per the instructions of the Animal Ethics protocols and the National Committee of Bio-Ethics, King Abdul-Aziz City of Science and Technology (KACST), Royal Decree No. M/59. All protocols hereby applied were reviewed and approved by the animal ethics committee of the Deanship of scientific research, King Faisal University, Saudi Arabia (Approval No: KFU-REC/2020-12-35). All the necessary paperwork for sample collection was obtained.

### Study period and location

This study was conducted from April to December 2019 in the Eastern region of Saudi Arabia.

### Sample collection from chickens and free-range wild birds

**Figure-2:**
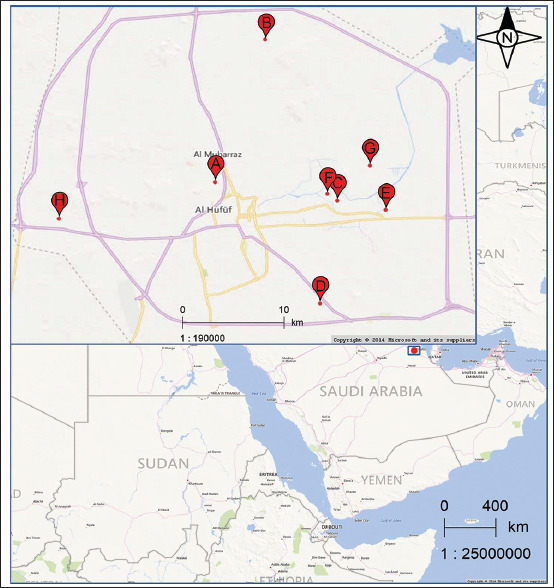
Map showing the geographic distribution of the outbreaks in the backyard chickens in the eastern region of Saudi Arabia [Source: www.maps.google.com].

Figure-2 shows the geographical distribution of the collected samples in Eastern Saudi Arabia.

Professional hunters captured 78 free-range birds from two locations (K and L), as shown in Figure-2. These birds included pigeons (*Columba livia*), doves (European turtledoves, *Streptopelia turtur*), lesser black-backed gulls (*Larus fucus*), and white-eared bulbuls (*Pycnonotus leucotis*), through nest trapping in locations near the backyards of the chickens studied (Table-1). The captured birds were collected early in the morning and transferred to the laboratory daily. Both chicken and wild bird samples were collected between November 2018 and April 2019. Briefly, whole-blood samples were collected from backyard chickens and captured wild birds from the lateral ulnar veins, as described by Hoysak and Weatherhead [21], and Owen [22]. The collected serum samples were stored at −20°C until further analysis.

**Table-1:** Results of the seroprevalence of the aMPV in wild birds around backyard chicken flocks.

Species	Tested	(+Ve)	(−Ve)
Pigeon (*Columba livia*)	46	0	46
Doves (European Turtle Dove, *Streptopelia turtur*)	18	0	18
Lesser Black-backed Gull (*Larus fucus*)	7	0	7
White-eared Bulbul (*Pycnonotus leucotis*)	7	0	7
Total	78	0	78

aMPV=Avian metapneumovirus

### Serological assay

The ID Screen^®^ aMPV Indirect kit (IDvet-France, catalog no.: MRIPOS-BIRD-RTU-B), which can detect aMPV subtypes A and B, was used for aMPV-related antibody detection. Enzyme-linked immunosorbent assay (ELISA) was performed according to the manufacturer’s instructions and as described by Franzo *et al*. [23]. Briefly, a 96-well plate containing the test and control specimens was prepared before transferring the samples to an ELISA microplate using a multichannel pipette to avoid differences in incubation times. A final dilution of 1:500 was achieved from each serum sample in the dilution buffer. A volume of 100 μL of prediluted samples, negative-control sera, or positive-control sera was added to the wells of microtiter plates precoated with the target virus antigen. After 30 min of incubation at 20°C, the plates were washed five times with wash buffer. Peroxidase-conjugated anti-chicken immunoglobulin G antibody was added to each well, followed by incubation for 30 min at 21°C. After three washes, substrate–chromogen solution was added to the plates, followed by incubation for 30 min at 21°C in the dark. Finally, stop solution was added to each well, and the color intensity was measured using a spectrophotometer (iMark™ Microplate Absorbance Reader, Bio-Rad, USA) at 450 nm. The test was considered valid if the ratio between the positive and negative controls’ optical density (OD) was higher than 3 per the manufacturer’s instructions. For each sample, the sample-to-positive-control ratio and antibody titer were calculated. We developed a scoring system by setting the cutoff at 396. We also developed a new scoring system for the antibody level in the tested sera (Table-1). This scoring system was based on the detected antibodies in the sera of the tested birds as follows: OD < 2 × cutoff (397–792), +; OD < 3 × cutoff (792–1188), ++; and OD > 3 × cutoff (>1188), +++.

### Statistical analysis

Kruskal–Wallis analysis was conducted to compare the prevalence of aMPV among the tested locations as described by Ostertagova *et al*. [24].

## Results

### Seroconversion of backyard domestic chickens to aMPV

Our results revealed that 74 of 294 backyard chicken sera were positive for aMPV in the ten test locations (A–J, Table-2). Conversely, all wild bird samples were negative for aMPV antibodies (Table-1).

**Table-2:** Results of the serological survey of the aMPV-related antibodies in backyard chickens.

Locations	Total No	Morbidity %	Mortality %	Total tested	Positive	% (+Ve)	Negative	(+)	(++)	(+++)
A	619	45	17	38	11	28.9	27	4	2	5
B	525	25	3	49	5	10.2	44	2	1	2
C	320	15	7	32	5	15.6	27	2	1	2
D	702	27	19	29	7	24.1	22	2	2	3
E	252	16	4	42	5	11.9	37	3	0	2
F	273	32	13	33	10	30.3	23	3	5	2
G	324	28	15	65	9	13.8	56	5	1	3
H	143	25	9	21	4	19	17	0	1	3
I	251	32	18	40	9	22.5	31	5	1	3
J	156	12	7	19	5	26.3	15	1	1	2
Total	3565			368	74	14.8	299	27	15	27

(−) OD<396, (+) = (<2×cutoff): 397–792, (++) = (<3×cutoff): 792–1188, (+++) = (>3×cutoff): >1188. aMPV=Avian metapneumovirus

### Domestic backyard chickens seroconverted to aMPV at various thresholds

Our data showed the seroconversion of some backyard chickens to aMPV at ten locations in Eastern Saudi Arabia (Figure-2 and Table-2). The overall seroprevalence among all areas was 14.8%. The highest positivity rates for aMPV antibodies were detected at F, A, J, D, and I (30.3%, 28.9%, 26.3%, 24%, and 22.5%, respectively, Table-2, Figure-2). Conversely, the positivity rates at H, C, G, E, and B were 19%, 15.6%, 13.8%, 11.9%, and 10.2%, respectively (Table-1, Figure-2).

## Discussion

MPV was first described in South Africa in 1978 and has subsequently spread to other parts of the world [25]. Avian metapneumovirus causes respiratory tract infections in chickens. It can also cause several clinical syndromes in turkeys, such as SHS. The severity of these clinical syndromes in birds varies according to the virus type [26]. Four aMPV, subtypes (A–D) based on G protein sequences have been used to categorize viral isolates. Based on these sequences, the phylogenetic trees clustered the European isolates (A, B, and D) of aMPV together. In contrast, the various lines of subtype C are closely related to human MPV [23]. Subtype B was reported to circulate widely in many European countries [23]. Some zero surveillance studies were conducted among chickens in various geographical locations globally, confirming the seroconversion of some of these chickens to aMPV [27–29]. Our data showed that some backyard chickens in ten locations across Eastern Saudi Arabia seroconverted to aMPV (Figure-1 and Table-2). No backyard chickens tested in this study were vaccinated against aMPV. Thus, the presence of antibodies in the sera of these animals strongly suggests recent exposure of these birds to natural aMPV infection. We developed a scoring system to measure the magnitude of the antibody titers in the sera of these backyard chickens. We found some variations in the response of these chickens to aMPV. We categorized these birds into three classes based on their antibody responses (+, ++, and +++), as shown in Table-2. The variations in the immune response and the antibody titers against aMPV in these chickens could be attributed to several factors. First, individual variations among these birds and some genetic factors could make some birds susceptible or resistant to this virus. Genetic factors play essential roles in the response of some chickens to viral diseases such as Marek’s disease and avian leucosis. This might also shape the vaccine response to these viruses [30].

**Figure-3:**
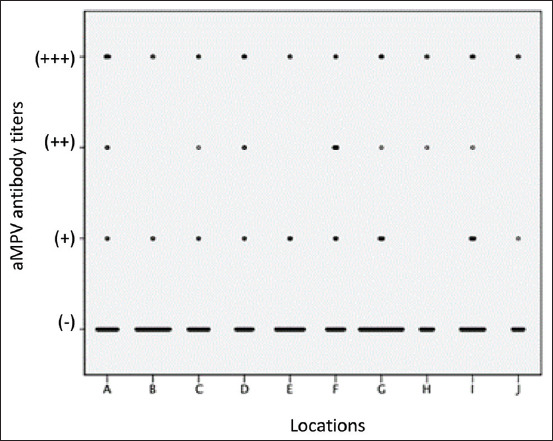
A two-dot plot also illustrates the distribution of the various data points. (-) indicate negative samples, (+) denotes low antibody titer, (++) moderate antibody titers, (++) strong antibody titer, and (+++) very strong antibody titer against aMPV.

There is another possibility that some birds are repeatedly exposed to aMPV, which increases the aMPV antibodies, resulting in a score of +++ (Figure-3). The increasing antibody titers after repeated infection are consistent with other avian viruses, such as infectious bronchitis virus [31].

Backyard chickens and wild birds play essential roles in transmitting and sustaining pathogens, particularly viral diseases, in domestic farm chickens. We previously reported several outbreaks of viral infections, including emerging and reemerging viral infections, in backyard chickens in many countries [6, 7, 32–34]. Avian metapneumovirus antibodies were detected in wild birds, such as sparrows and pigeons, in several regions, including Europe and North America [23, 29, 35–37].

This is in contrast to this study. In this study, we tested sera from hunted wild birds that were in close contact with backyard chickens. Although our data did not detect aMPV in the tested wild birds, this does not eliminate the potential roles of these birds in the transmission of viral diseases of chickens, including aMPV. The lack of detection of aMPV antibodies in the sera of these wild birds could be attributed to the small number of tested samples or the specificity of these kits for aMPV detection in wild birds. Further large-scale studies are needed to explore the roles of backyard chickens and wild birds in MPV transmission.

Several studies have reported the incidence of aMPV in several countries with a noticeably high incidence rate. Based on the antibody titer, the incidence was 73.1% in chickens in Korea [38]. The rates in Bangladesh and Tamil Nadu state in India were 53.29% and 34.02%, respectively [39, 40]. In Ukraine, 78% of turkeys and 100% of “Birkivska Barvysta” hens were aMPV carriers [41]. We found that the incidence rate in the region we studied in Saudi Arabia was significantly lower than the global average. Further research is planned to characterize the virus on a molecular basis, enabling comparisons of virus sequences across different continents. However, the nature of the virus and the resistance of local chicken breeds might explain the observed low incidence rate.

The source of potential virus attraction in our examined chickens is a plausible finding. In a previous study, aMPV was found to cross the barriers of species specificity. The researchers compared two strains of AMPV-C: one from a turkey in the United States (turkey AMPV-C) and one from a duck in France (duck AMPV-C). AMPV-A, AMPV-B, turkey AMPV-C, and AMPV-D were well adapted to Galliformes, particularly turkeys, although chickens displayed milder symptoms and a different seroconversion and transmission pattern. For the first time, chickens were demonstrated to be vulnerable to AMPV-D and incapable of transferring AMPV-A to their contacts. When exposed to duck AMPV-C, chickens and turkeys seroconverted and tested positive for the virus. Turkeys became seropositive after exposure to duck AMPV-C, demonstrating that this virus can be transmitted horizontally between non-palmed species for the first time. Although no viral RNA was found in ducks, chickens, or turkeys, AMPV-C was isolated. Furthermore, turkey AMPV-C was proven to be extremely specific for turkeys, and it was effectively isolated from chickens despite the absence of detectable viral RNA. These data suggest that adaptation to a “non-conventional host” favors viral genomic sequence variants that differ from the original strain [31]. More research is needed to isolate and compare the genomic sequences of aMPV from domestic birds such as ducks and turkeys from locally affected locations and assign genetic differences and overlapping cross-reactivity across chicken, turkey, and duck isolates.

Wild Canadian geese collected in Minnesota and sentinel ducks deployed near an infected commercial turkey farm tested positive for avian pox virus, indicating the potential role of free-ranging birds in aMPV transmission [42]. The method used in our assay detects aMPV subtypes A and B. These subtypes might not be circulating among wild birds, and thus, they need to be reexamined for possible infection with subtypes C and D.

## Conclusion

Avian metapneumovirus is a new respiratory virus found in chickens; consequently, knowledge about its global distribution is limited. Chickens kept as pets in the backyard can play a significant role in the spread and persistence of some avian viruses. The primary objective of this research was to examine the exposure of hens kept in backyards and wild birds that frequented the areas where these chickens lived. To accomplish this, we collected 368 serum samples from ten distinct backyard hen farms in Eastern Saudi Arabia. We obtained 78 serum samples from the wild Columbidae species living in these areas. According to our findings, 74/368 birds tested positive for aMPV. The levels of antibodies in these birds were inconsistent. There was no seroconversion for aMPV among the sampled wild birds. Exposure of these domestic backyard chickens was evidenced by their seroconversion to aMPV. In other words, this is conclusive proof that this seroconversion was caused by a recent natural infection. The detection of aMPV antibodies in some unvaccinated domestic backyard chickens strongly suggests their exposure to a natural infection by these viruses.

## Authors’ Contributions

AIAM and MGH: Conceived the idea. AIAM: Funding and project management; AIAM, JH, MK, AAGA, BF, and MGH: Sample collection, laboratory testing, data interpretations, and wrote the manuscript. All authors have read, reviewed and approved the final manuscript.

## Data Availability

The supplementary data can be available from the corresponding author on a reasonable request.
